# Knowledge and attitudes of primary care physicians in the management of patients at risk for cardiovascular events

**DOI:** 10.1186/1471-2296-9-42

**Published:** 2008-07-08

**Authors:** Hamidreza Doroodchi, Maziar Abdolrasulnia, Jill A Foster, Elyse Foster, Mintu P Turakhia, Kimberly A Skelding, Kiran Sagar, Linda L Casebeer

**Affiliations:** 1 Outcomes, Inc., Birmingham, USA; 2 Department of Clinical Medicine & Anesthesia, University of California, San Francisco, USA; 3 Department of Medicine, University of California, San Francisco, USA; 4 Department of Medicine, Geisinger Medical Center, Danville, USA; 5 Department of Medicine, University of Wisconsin Madison, Whitefish Bay, USA

## Abstract

**Background:**

Adherence to clinical practice guidelines for management of cardiovascular disease (CVD) is suboptimal. The purposes of this study were to identify practice patterns and barriers among U.S. general internists and family physicians in regard to cardiovascular risk management, and examine the association between physician characteristics and cardiovascular risk management.

**Methods:**

A case vignette survey focused on cardiovascular disease risk management was distributed to a random sample of 12,000 U.S. family physicians and general internists between November and December 2006.

**Results:**

Responses from a total of 888 practicing primary care physicians who see 60 patients per week were used for analysis. In an asymptomatic patient at low risk for cardiovascular event, 28% of family physicians and 37% of general internists made guideline-based preventive choices for no antiplatelet therapy (p < .01). In a patient at high risk for cardiovascular event, 59% of family physicians and 56% of general internists identified the guideline-based goal for serum fasting LDL level (< 100 mg/dl). Guideline adherence was inversely related to years in practice and volume of patients seen. Cost of medications (87.7%), adherence to medications (74.1%), adequate time for counseling (55.7%), patient education tools (47.1%), knowledge and skills to recommend dietary changes (47.8%) and facilitate patient adherence (52.0%) were cited as significant barriers to CVD risk management.

**Conclusion:**

Despite the benefits demonstrated for managing cardiovascular risks, gaps remain in primary care practitioners' management of risks according to guideline recommendations. Innovative educational approaches that address barriers may facilitate the implementation of guideline-based recommendations in CVD risk management.

## Background

Cardiovascular disease (CVD) remains the number one cause of death in the United States. Numerous advances in medical therapy and diagnostics have occurred for prevention and treatment of CVD. In order to standardize clinical practice and simplify clinical decision making in reducing CVD risk, clinical guidelines have been published and are available: the American Heart Association/American College of Cardiology [1], National High Blood Pressure Education Program committee (JNC 7) [2], and National Cholesterol Education Panel (ATP III) [3]. Yet, despite efforts to reduce the risk of CVD among at risk Americans, recent observation and survey studies show that considerable gaps in knowledge and application of guideline recommendations for risk reduction remain [4-9]. A key factor in proper CVD risk management is accurate risk assessment; however inconsistencies among current methods for calculating risk [10] and the perception of risk among health care providers contribute to challenges in risk assessment [7,11]. Results from several studies demonstrate that treatment strategies have not achieved blood pressure and lipid profile goals in women, African Americans, Hispanic Americans, young people, and patients with co-morbidities [12-20]. While the benefits of reducing blood pressure in a hypertensive patient are obvious [2], multiple studies have shown that more than 40% are satisfied with blood pressures higher than the recommended goal [21-24]. The objectives of this study were to: 1) assess the knowledge, attitudes and identify barriers to optimal CVD prevention among US primary care physicians; 2) examine the association between physician characteristics and their knowledge and attitudes.

## Methods

We performed a case vignette survey of U.S. primary care physicians between November and December 2006. Surveys were distributed by fax and electronic mail to a random sample of 12,000 board-certified family physicians and general internists. Participants were offered a $20 gift card to complete the study. For this study, respondents who saw 60 or more patients per week were considered active physicians. Of 1,025 responses (8.5% response rate), 888 met the criteria as an active family physician or general internist and saw 60 or more patients per week.

### Survey development

Four physician authors (E. F., M. T., K. S., and K. S.) developed a series of case vignettes designed to examine current practice patterns of primary care physicians in CVD risk assessment and management. Initially, the scientific literature was reviewed to examine known and suspected gaps between actual physician practice and evidence-based guidelines concerning the management of cardiovascular risk in order to guide survey development. Based on the evidence and guidelines, a series of case vignettes were developed. This approach has been shown to be an effective and cost-efficient method for measuring physicians' clinical decision making [25-28]. The case vignettes described scenarios of low- and high-risk patients and included information about the patient's age, gender, ethnicity/race, smoking status, total cholesterol level, LDL-cholesterol level, HDL-cholesterol level, triglycerides, blood pressure, treatment for hypertension, family history and personal history of heart disease, or diabetes mellitus. Physicians were queried as to the patient's cardiovascular risk, LDL goal, and dietary and therapeutic recommendations. In addition to the clinical vignettes, survey items were designed to measure physician confidence and perceived barriers to optimal CVD prevention and management using a 5 and 10 point Likert scale as well as learning preferences and demographic characteristics. (Additional file 1)

### Statistical analysis

Chi-square (χ^2^) tests were performed for categorical data, while t-tests were used on normally distributed continuous data. These tests were conducted using a level of statistical significance of .05. All analyses were conducted using SPSS V.15.0 (SPSS, Inc. Chicago, IL).

## Results

### Physician demographics

Descriptive statistics of physician characteristics are presented in Tables 1 and 2 with reporting of proportions, means, and standard deviations. A total of 888 primary care physician survey responses were eligible for this study: 562 family physicians and 326 general internists. The average years-in-practice for respondents was 18.8 (SD = 10.3) years for family physicians and 18.0 (SD = 9.3) years for general internists. The sample was comprised primarily of male physicians (family physicians 78%; general internists 78%), those with a MD degree (family physicians 85%; general internists 97%), those in private practice (family physicians 87%; general internists 90%) and those who practice in an urban or suburban area (family physicians 68%; general internists 90%). On average, family physicians saw 38 (SD = 21.9) patients per week with hypertension/dyslipidemia and general internists saw 45 (SD = 23.9) patients per week with hypertension/dyslipidemia. Compared to the characteristics of U.S. family physicians and general internists identified by the Physician Masterfile of the American Medical Association (AMA), this sample was representative of US primary care physicians in terms of years-in-practice, gender, and degree.

**Table 1:** Demographics of family physician respondents compared to American Medical Association (AMA) family physicians

	Family Physicians (N = 562)	AMA
Years in practice: Mean (SD)	18.75 (10.3)	22.5 (12.2)

Gender: % (N)		

Male	77.8% (367)	70.2% (63,228)

Female	22.2% (105)	29.8% (26,874)

Degree: % (N)		

MD	85.4% (480)	83.1% (74,892)

DO	14.6% (82)	16.9% (15,210)

Private practice: % (N)	86.8% (414)	--

Practice location: % (N)		

Urban	20.9% (100)	--

Suburban	46.6% (223)	--

Rural	32.6% (156)	--

Patients with hypertension/dyslipidemia seen per week: Mean (SD)	37.8 (21.9)	--

**Table 2:** Demographics of general internist respondents compared to American Medical Association (AMA) general internists

	General Internists (N = 326)	AMA
Years in practice: Mean (SD)	18.0 (9.3)	22.3 (11.1)

Gender: % (N)		

Male	78.1% (228)	68.9% (58,799)

Female	21.9% (64)	31.1% (26,553)

Degree: % (N)		

MD	97.2% (317)	95.2% (81,284)

DO	2.8% (9)	4.8% (4,068)

Private practice: % (N)	89.7% (261)	--

Practice location: % (N)		

Urban	37.9% (110)	--

Suburban	51.7% (150)	--

Rural	10.3% (30)	--

Patients with hypertension/dyslipidemia seen per week: Mean (SD)	45.0 (23.9)	--

### Risk factor management in low and high risk patients

In an asymptomatic 45-year-old woman with metabolic syndrome who is at a 10-year low risk for CVD (Framingham 1%), 28% of family physicians and 37% of general internists chose the guideline recommendations for no antiplatelet therapy to prevent myocardial infarction (p < 0.01). The majority (Family physicians 65%, General internists 54%) indicated that they would prescribe aspirin 81 mg daily for such a patient to reduce the risk of myocardial infarction. When asked about dyslipidemia pharmacotherapy recommendations for this same low risk patient, 51% of all primary care respondents indicated no lipid lowering pharmacotherapy in accordance with guideline recommendations; however, 41% selected a statin. For the low risk patient case, 24% of all primary care physicians selected no specific dietary fat avoidance as long as it does not exceed 30% of total intake; however, 62% selected guideline-based dietary recommendations to avoid trans fatty acids.

Two cases designating patients with high CVD risk were also presented. For a 50-year-old male with high CVD risk, 59% of family physicians and 56% of general internists identified the Adult Treatment Panel III (ATP III) [3] guideline-based goal for fasting serum LDL level (< 100 mg/dl); 12% overall would accept a higher LDL level (< 130 mg/dl) while 30% chose a target which is recommended for very high risk patients (< 70 mg/dl) [29]. For a 78-year-old female with high CVD risk, 72% of family physicians and 76% of general internists chose the recommended option to initiate lifestyle and dietary modification and treat with both a thiazide diuretic and a statin. For the same patient at high risk for CVD and no overt CHD symptoms, 48% of family physicians and 49% of general internists were in concordance with guidelines to order a stress test if she develops symptoms of chest pain, shortness of breath or atypical angina (Table 3).

**Table 3:** Differences in practice patterns of family physicians and general internist respondents in managing CVD* risk

	Family Physicians (N = 562)	General Internists (N = 326)	p-value
**Antiplatelet therapy for prevention of myocardial infarction in a 45-year-old ** **asymptomatic woman with one BP reading of 145/90 mm Hg, BMI 28 kg/m2,** **LDL 125 mg/dL, HDL 55 mg/dL, TG 200 mg/dL, and normal glucose**			

Aspirin 100 mg every other day	0.9%	0.9%	< 0.01
Aspirin 81 mg daily	65.6%	54.3%	
Aspirin 325 mg daily	4.8%	5.8%	
Clopidogrel 75 mg daily	0.9%	2.5%	
No antiplatelet therapy**	27.8%	36.5%	

**Dyslipidemia pharmacotherapy recommendation for a 45-year-old ** **asymptomatic woman with one BP reading of 145/90 mm Hg, BMI 28 kg/m2,** **LDL 125 mg/dL, HDL 55 mg/dL, TG 200 mg/dL, and normal glucose**			

Atorvastatin 10 mg every evening	42.4%	41.0%	0.84
Ezetimide 10 mg daily	2.5%	4.3%	
Niacin 500 mg twice daily	3.9%	3.7%	
No specific therapy for dyslipidemia**	51.2%	50.9%	

**Dietary recommendation (avoiding fat) for a 45-year-old asymptomatic woman ** **with one BP reading of 145/90 mm Hg, BMI 28 kg/m2, LDL 125 mg/dL, HDL 55 ** **mg/dL, TG 200 mg/dL, and normal glucose**			

Trans fatty acids**	62.5%	61.8%	0.91
Polyunsaturated fats	9.6%	9.2%	
Mono-unsaturated fats	3.2%	5.8%	
No specific fat as long as it does not exceed 30% of total intake	24.6%	23.1%	

**LDL Goal for a 50-year-old asymptomatic man, negative family history of ** **premature CHD, BP 170/94 mm Hg, BMI 26 kg/m2, total cholesterol 210 mg/dL,** **LDL 130 mg/dL, HDL 36 mg/dL, TG 256 mg/dl, Fasting glucose 140 mg/dL, and ** **normal exercise stress test**			

LDL < 130 mg/dl	11.8%	12.3%	0.36
LDL < 100 mg/dl**	59.0%	56.0%	
LDL < 70 mg/dl	29.2%	31.7%	

**Hypertension and dyslipidemia management for a new asymptomatic 78-year- ** **old female patient with questionable history of diabetes, BP 159/78 mm Hg, BMI** **29 kg/m2, LDL 199 mg/dL, TG 479 mg/dL, and HbA1c of 6.0%**			

Lifestyle and dietary modification	6.8%	8.0%	0.11
Lifestyle and dietary modification and treatment with a thiazide diuretic	6.6%	5.2%	
Lifestyle modification and treatment with a statin	14.9%	10.2%	
Lifestyle and dietary modification and treatment with both a thiazide diuretic and a statin**	71.7%	76.5%	

**Approach to stress testing for a new asymptomatic 78-year-old female patient ** **with questionable history of diabetes, BP 159/78 mm Hg, BMI 29 kg/m2, LDL** **199 mg/dL, TG 479 mg/dL, and HbA1c of 6.0%**			

I would order one as a follow up to today's visit	39.6%	33.0%	0.88
I would order one today and yearly thereafter	5.6%	10.5%	
I would order a test if she develops symptoms of chest pain, shortness of breath or atypical angina**	48.1%	48.8%	
I would not order a stress test on this elderly woman	6.7%	7.7%	

*CVD, cardiovascular disease, CHD, coronary heart disease

**Evidence-based guideline choice. T-test was performed comparing the two groups and their responses to the evidence-based guideline choice.

### Demographics characteristics and practice patterns

Primary care physicians who have been in practice for 10 years or less were significantly more likely to make practice choices in accordance with guideline recommendations to manage low and high risk patients than physicians who have been in practice for more than 10 years (.58 vs. .52, p < 0.01). Primary care physicians who estimate seeing a small number of patients with hypertension and dyslipidemia (25% or less) were significantly more likely to make practice choices in accordance to guideline recommendations to manage low and high risk patients than physicians who estimate seeing a large number of patients with hypertension and dyslipidemia (greater than 25%) (.58 vs. .52, p < 0.01). Geographic location did not have any association with practice choice and guideline adherence (Table 4).

**Table 4:** Association between demographic characteristics and physician practice patterns in managing CVD* patients

	**Family Physicians**	**p-value**	**General Internists**	**p-value**	Overall*	**p-value**
**Years in practice**						

10 or less	.56 (n = 155)	0.03	.60 (n = 91)	0.02	.58 (n = 246)	< 0.01

Greater than 10	.52 (n = 406)		.53 (n = 235)		.52 (n = 641)	

**Percent of patients ** **with hypertension ** **or dyslipidemia**						

25% or less per week	.57(n = 196)	< 0.01	.61 (n = 64)	0.02	.58 (n = 260)	< 0.01

Greater than 25% per week	.51 (n = 363)		.53 (n = 262)		.52 (n = 625)	

**Geographic location**						

Urban/Suburban	.53 (n = 323)	0.79	.55 (n = 260)	0.98	.54 (n = 583)	0.54

Rural	.52 (n = 156)		.55 (n = 30)		.53 (n = 186)	

*CVD, cardiovascular disease

†Overall refers to the combined results of family physicians and general internists

‡Scores calculated as mean correct identification to clinical case questions

### Barriers to optimal CVD management

Practice and patient barriers are listed in Table 5. Overall, cost of medications was rated as the most significant barrier to CVD risk management (87.7%). Additionally the number of medications needed for adequate blood pressure control and patient adherence were also indicated as significant barriers to CVD patient management (75.1% and 74.1%). Nearly 50% in both groups also cited adequate time, patient education tools and knowledge, and skills to recommend dietary changes and facilitate patient adherence as a significant barrier for them and their staff.

**Table 5:** Perceived barriers for managing CVD patients

	**Family Physicians**	**General Internists**	**Overall**
Adverse effects of drugs	50.4%	52.3%	51.1%

Patient adherence	73.7%	74.1%	73.8%

Presence of co-morbid conditions	59.6%	56.3%	58.4%

Cost of medications	89.4%	87.7%	88.8%

Number of drugs needed for adequate blood pressure control	74.0%	75.1%	74.4%

Patient understanding of treatment goals	53.8%	51.4%	52.9%

Adequate time to address lifestyle issues with patients	52.2%	55.4%	53.4%

Adequate patient-education tools regarding lifestyle issues	44.0%	47.1%	45.1%

Knowledge and skills to provide dietary recommendations	46.2%	47.8%	46.9%

Knowledge and skills to facilitate patient adherence	44.7%	52.0%	47.4%

Each item was rated on a 1–5 Likert scale (1 = not a significant barrier, 5 = significant barrier).

Percentage reported represents those who indicated a 4 or 5 on the scale.

CVD, cardiovascular disease

Overall refers to the combined results of family physicians and general internists

### Information sources and preferences

Thirty-nine percent of survey respondents considered randomized controlled trials or meta-analyses as the minimum level of evidence acceptable as the basis for determining an appropriate treatment regimen, while 23% considered clinical practice guidelines their minimum standard. Survey respondents selected clinical practice guidelines as the most important tool in helping them provide optimal care to their patients.

## Discussion

The main findings from this study were that guideline concordance in managing low and high risk CVD patients by primary care physicians significantly varied in treatment and dietary approach, and managing patients according to guidelines was associated with years in practice and volume of patients. For a low-risk female patient about one third of primary care physicians followed guidelines to not initiate antiplatelet therapy. These findings are similar to other studies reporting differences in management approach by risk level assessment by physicians [7,21]. In a study of 300 primary care physicians, Mosca et al. found that 32% will prescribe aspirin for a low-risk patient [7]. Additionally, for a 50-year-old high risk patient 40% did not indicate a guideline recommended LDL goal in accordance with his risk level. Results of recent clinical trials, however, suggest that the lower the serum LDL-cholesterol level, the more the benefits in preventing cardiovascular events [30,31]. The recognition and appropriate management of low- and high-risk patients is critical especially by primary care physicians because in many cases, especially in underserved areas, they serve as the only source of care.

In terms of dietary recommendations, our study found that over one-third of primary care physicians failed to recommend reducing trans fatty acid intake for CVD prevention in a low risk patient. This finding is consistent with other studies showing a lack of guideline-based dietary recommendations by primary care physicians [7]. A plausible cause for this finding may be that nearly 50% of physicians indicated that their and their staff's knowledge and skills to provide dietary recommendations is a significant barrier in their practice. Innovative educational interventions and practical screening tools to calculate CVD risk may be useful to overcoming this barrier.

It is noteworthy that two main characteristics of primary care physicians were associated with greater guideline concordance. Physicians who have been in practice for 10 years or less and physicians who managed a small number of patients with hypertension and dyslipidemia (25% or less) were significantly more likely to make practice choices in accordance to guidelines. These findings are consistent with results of a systematic review conducted by Choudhry and colleagues which suggested an inverse relationship between years in practice and the quality of care provided [32]; other studies suggest that older physicians are more likely to be aware and incorporate guidelines to practice [11].

Reverse relationship between guideline adherence and patient volume is especially concerning in that physicians' who see a greater percentage of hypertensive and dyslipidemic patients are not providing care according to standards. Younger physicians are more likely to adhere to guidelines than more experienced physicians. A plausible explanation for this finding is that it may be more difficult for older physicians to overcome previous practice inertia [24,33]. A review by Cabana and colleagues suggest a variety of other reasons (not related to physician's age) why physicians may not adhere to guidelines including a lack of knowledge of the guidelines, disagreement with the evidence, and lack of expectations that adherence will result in better patient outcomes [33].

Although clinical practice guidelines were identified by approximately one-third of survey respondents as the most important tool for delivering optimal care to their patients, only a quarter of respondents accept guidelines as the minimum level of evidence for determining an appropriate treatment regimen. Close to 40% indicated that they would accept a level of evidence that is below level A randomized controlled trial or meta-analysis. This, especially in the context where a substantial number of respondents set very aggressive lipid goals that are in line with recent trial data, could very well indicate a need for more frequent revisions of clinical practice guidelines as new data emerges. More frequent updates of the guidelines could increase physician confidence in the recommendations and improve physician adherence.

Finally, an interesting finding in our study was that 25% of primary care physicians selected CME activities as the most important tool in helping them improve patient care. CME was rated above clinical practice guidelines in keeping physicians up-to-date. Physicians expressed that they would prefer CME content that is patient-centered and that provides strategies for daily practice, rather than information on trial methodology and data.

There are several limitations to this study. First, this study used a survey as a surrogate measure of primary care physicians' knowledge and attitudes that was self-reported. However, the use of case vignettes has been shown to provide good insight into physicians' actual practice patterns [25-28]. Second, only four clinical scenarios were used which do not cover the full spectrum of cardiovascular risk. We specifically examined recognition of cardiovascular risk, goal setting, and treatment recommendations. Future studies are needed to examine more specific areas of cardiovascular risk recognition and if practice choices will vary according to other relevant variables such as patients' health insurance status, patient gender and race, and socioeconomic status that may be strong determinants of clinical choices. Additionally, respondents were given a small honorarium to complete the study, which could influence physician participation rates and responses. The cross-sectional design of the study does not allow for causal inferences to be drawn and future study designs such as cohort and longitudinal designs are needed. Finally, the majority of practitioners were in private practice and the impact of a managed care environment on adherence to guidelines was not evaluated. Managed care restrictions and penetration may influence practice choices and attitudes of physicians in how they treat patients.

## Conclusion

In conclusion, despite the benefits demonstrated for managing cardiovascular risks, gaps remain in primary care practitioners' management of risks according to guideline recommendations. Innovative educational approaches are needed to address barriers, and target specific groups of physicians to facilitate the implementation of guideline-based recommendations in CVD management.

## Competing interests

The authors declare that they have no competing interests.

## Authors' contributions

HD participated in the design of the study and drafted the manuscript. EF, KS, MT, KS participated in the development of the survey instrument and reviewed the manuscript. MA performed the statistical analysis and drafted the methods section of the manuscript. JF participated in the review of the manuscript and the writing of the discussion section. LC participated in the design of the study. All authors read and approved the final manuscript.

## Pre-publication history

The pre-publication history for this paper can be accessed here:



## Supplementary Material

Additional file 1 Appendix A. Click here for file

## References

[B1] American Heart Association/American College of Cardiology Guidelines. American Heart Association and American College of Cardiology Websites. http://www.americanheart.org/presenter.jhtml?identifier=3004572.

[B2] Chobanian AV, Bakris GL, Black HR, Cushman WC, Green LA, Izzo JL, Jones DW, Materson BJ, Oparil S, Wright JT, Roccella EJ, National Heart, Lung, and Blood Institute Joint National Committee on Prevention, Detection, Evaluation, and Treatment of High Blood Pressure, National High Blood Pressure Education Program Coordinating Committee (2003). The Seventh Report of the Joint National Committee on Prevention, Detection, Evaluation, and Treatment of High Blood Pressure: the JNC 7 report. JAMA.

[B3] Expert Panel on Detection, Evaluation, and Treatment of High Blood Cholesterol in Adults (2002). Third report of the National Cholesterol Education Program (NCEP) Expert Panel on Detection, Evaluation, and Treatment of High Blood Cholesterol in Adults (Adult Treatment Panel III): Final Report. Circulation.

[B4] Asch SM, Kerr EA, Keesey J, Adams JL, Setodji CM, Malik S, McGlynn EA (2006). Who is at greatest risk for receiving poor-quality healthcare?. N Engl J Med.

[B5] Davidson MH, Maki KC, Pearson TA, Pasternak RC, Deedwania PC, McKenney JM, Fonarow GC, Maron DJ, Ansell BJ, Clark LT, Ballantyne CM (2005). Results of the National Cholesterol Education Program Evaluation Project utilizing novel E-technology (NEPTUNE) II survey and implications for treatment under the recent NCEP Writing Group recommendations. Am J Cardiol.

[B6] Ma J, Sehgal NL, Ayanian JZ, Stafford RS (2005). National trends in statin use by coronary heart disease risk category. PLoS Med.

[B7] Mosca L, Linfante AH, Benjamin EJ, Berra K, Hayes SN, Walsh BW, Fabunnmi RP, Kwan J, Mills T, Simpson SL (2005). National study of physician awareness and adherence to cardiovascular disease prevention guidelines. Circulation.

[B8] Muntner P, He J, Roccella EJ, Whelton PK (2002). The impact of JNC-VI guidelines on treatment recommendations in the US population. Hypertension.

[B9] Rosamond W, Flegal K, Friday G, Furie K, Go A, Greenlund K, Haase N, Ho M, Howard V, Kissela B, Kittner S, Lloyd-Jones D, McDermott M, Meigs J, Moy C, Nichol G, O'Donnell CJ, Roger V, Rumsfeld J, Sorlie P, Steinberger J, Thom T, Wasserthiel-Smoller S, Hong Y, American Heart Association Statistics Committee and Stroke Statistics Subcommittee (2007). Heart Disease and Stroke Statistics–2007 Update: A Report from the American Heart Association Statistics Committee and Stroke Statistics Subcommittee. Circulation.

[B10] Assmann G (2005). Calculating global risk: the key to intervention. Eur Heart J Suppl.

[B11] Christian AH, Mills T, Simpson SL, Mosca L (2006). Quality of cardiovascular disease preventive care and physician/practice characteristics. J Gen Intern Med.

[B12] Correa-de-Araujo R, Stevens B, Moy E, Nilasena D, Chesley F, McDermott K (2006). Gender differences across racial and ethnic groups in the quality of care for acute myocardial infarction and heart failure associated with comorbidities. Womens Health Issues.

[B13] Goff DC, Bertoni AG, Kramer H, Bonds D, Blumenthal RS, Tsai MY, Psaty BM (2006). Dyslipidemia prevalence, treatment, and control in the Multi-Ethnic Study of Atherosclerosis (MESA): gender, ethnicity, and coronary artery calcium. Circulation.

[B14] Hajjar I, Miller K, Hirth V (2002). Age-related bias in the management of hypertension: a national survey of physicians' opinions on hypertension in elderly adults. J Gerontol A Biol Sci Med Sci.

[B15] Henderson SO, Bretsky P, DeQuattro V, Henderson BE (2003). Treatment of hypertension in African Americans and Latinos: the effect of JNC VI on urban prescribing practices. J Clin Hypertens (Greenwich).

[B16] Massing MW, Foley KA, Carter-Edwards L, Sueta CA, Alexander CM, Simpson RJ (2004). Disparities in lipid management for African Americans and Caucasians with coronary artery disease: a national cross-sectional study. BMC Cardiovasc Disord.

[B17] Mehta SS, Wilcox CS, Schulman KA (1999). Treatment of hypertension in patients with comorbidities: results from the study of hypertensive prescribing practices (SHyPP). Am J Hypertens.

[B18] Oparil S, Miller AP (2005). Gender and blood pressure. J Clin Hypertens (Greenwich).

[B19] Raji MA, Kuo YF, Salazar JA, Satish S, Goodwin JS (2004). Ethnic differences in antihypertensive medication use in the elderly. Ann Pharmacother.

[B20] Sonel AF, Good CB, Mulgund J, Roe MT, Gibler WB, Smith SC, Cohen MG, Pollack CV, Ohman EM, Peterson ED, CRUSADE Investigators (2005). Racial Variations in Treatment and Outcomes of Black and White Patients With High-Risk Non-ST-Elevation Acute Coronary Syndromes: Insights From CRUSADE. Circulation.

[B21] Hyman DJ, Pavlik VN (2000). Self-reported hypertension treatment practices among primary care physicians: blood pressure thresholds, drug choices, and the role of guidelines and evidence-based medicine. Arch Intern Med.

[B22] World Hypertension League calls for urgent action to get more hypertensive patients to goal. Hypertension News.

[B23] Borzecki AM, Oliveria SA, Berlowitz DR (2005). Barriers to hypertension control. Am Heart J.

[B24] Phillips LS, Branch WT, Cook CB, Doyle JP, El-Kebbi IM, Gallina DL, Miller CD, Ziemer DC, Barnes CS (2001). Clinical Inertia. Ann Intern Med.

[B25] Peabody JW, Luck J, Glassman P, Dresselhaus TR, Lee M (2000). Comparison of vignettes, standardized patients, and chart abstraction: a prospective validation study of 3 methods for measuring quality. JAMA.

[B26] Peabody JW, Luck J, Glassman P, Jain S, Hansen J, Spell M, Lee M (2004). Measuring the quality of physician practice by using clinical vignettes: a prospective validation study. Ann Intern Med.

[B27] Luck J, Peabody JW, Lewis BL (2006). An automated scoring algorithm for computerized clinical vignettes: evaluating physician performance against explicit quality criteria. Int J Med Inform.

[B28] Veloski, Jon , Tai Stephen, Evans AdamS, Nash DavidB (2005). Clinical vignette-based surveys: a tool for assessing physician practice variation. Am J Med Qual.

[B29] Grundy SM, Cleeman JI, Merz CN, Brewer HB, Clark LT, Hunninghake DB, Pasternack RC, Smith SC, Stone NJ, National Heart, Lung, and Blood Institute, American College of Cardiology Foundation, American Heart Association (2004). Implications of recent clinical trials for the National Cholesterol Education Program Adult Treatment Panel III guidelines. Circulation.

[B30] Abookire SA, Karson AS, Fiskio J, Bates DW (2001). Use and monitoring of "statin" lipid-lowering drugs compared with guidelines. Arch Intern Med.

[B31] Heart Protection Study Collaborative Group (2002). MRC/BHF Heart Protection Study of cholesterol lowering with simvastatin in 20,536 high-risk individuals: a randomized placebo-controlled trial. Lancet.

[B32] Choudhry NK, Fletcher RH, Soumerai SB (2005). Systematic review: the relationship between clinical experience and quality of health care. Ann Intern Med.

[B33] Cabana MD, Rand CS, Powe NR, Wu AW, Wilson MH, Abboud PA, Rubin HR (1999). Why don't physicians follow clinical practice guidelines? A framework for improvement. JAMA.

