# Supplementary material for: Knowledge and attitudes of primary care physicians in the management of patients at risk for cardiovascular events
Source: BMC Fam Pract. 2008 Jul 8;9:42. doi: 10.1186/1471-2296-9-42 (PMC2474612; doi:10.1186/1471-2296-9-42)
Supplement: Additional file 1 — Appendix A. [file 1471-2296-9-42-S1.doc]

**Appendix A**

# Case # 1: A 45 year old Caucasian woman presents for an initial routine primary care visit. She is asymptomatic but expresses concern regarding her risk for coronary artery disease because her mother recently suffered a myocardial infarction at age 72. She walks her dog daily for 30 minutes and attends a mat pilates class once a week. She does not smoke and has 3-5 glasses of wine/weekly. Her menses are regular. On physical examination, her blood pressure is 145/90, HR 72, weight 145 lbs, Height 64”, BMI = 28. The remainder of her physical examination is normal. Laboratory values reveal a fasting blood sugar of 94, Total cholesterol of 220, LDL of 125, HDL of 55.

1. Which of the following anti-platelet therapies would you recommend to reduce her risk of myocardial infarction?

| ❑ | Aspirin 100 mg every other day |
| --- | --- |
| ❑ | Aspirin 81 mg daily |
| ❑ | Asprin 325 mg daily |
| ❑ | Clopidogrel 75 mg daily |
| ❑ | No antiplatelet therapy |

1. Which therapy would you recommend for dyslipidemia?

| ❑ | Atorvastatin 10 mg every evening |
| --- | --- |
| ❑ | Ezetimide 10 mg daily |
| ❑ | Niacin 500 mg twice daily |
| ❑ | No specific therapy for dysplipidemia |

1. **You recommend initation of antihypertensive therapy, but she would like to try diet and exercise first. You suggest that she try to lose 20 lbs with a 1500 calorie diet; you recommend that sh**e avoid:

| ❑ | Trans fatty acids |
| --- | --- |
| ❑ | Polyunsaturated fats |
| ❑ | Mono-unsaturated fats |
| ❑ | No specific fat as long as it does not exceed 30% of total intake |

**Case #2:**  A 68-year old woman is referred to you for management of hypertension. She was diagnosed with stage II hypertension sixteen months ago with an initial blood pressure of 175/96. Although her primary care physician has prescribed a regimen of a diuretic, beta blocker, and ACE inhibitor, but her systolic blood pressures have remained above 160 mmHg. She has no symptoms of angina or heart failure. You ask her to review her medications, but she says she forgot to bring her prescription bottles. She says she is taking “two pills every morning and two or three pills at night for her blood pressure.” A review of her primary care physician’s note indicates that she is prescribed hydrochlorothiazide 25 mg daily, metoprolol 150 mg bid, and captopril 25 mg three times daily. She is on four additional medications for rheumatoid arthritis and chronic pain.

On physical examination, blood pressure is 182/96 mm Hg, heart rate is 78. Cardiac examination reveals a non-displaced, nonsustained point of maximal impulse. There is a prominent fourth heart sound but no murmurs. The rest of the exam is unremarkable.

1. **What is the most likely cause of her persistent hypertension? (select only one)**

| ❑ | Renal artery stenosis |
| --- | --- |
| ❑ | Pseudohypertension from arterial sclerosis |
| ❑ | Poor adherence to medications |
| ❑ | “White Coat” hypertension |

1. **Which of the following contributes most to poor medication adherence in the elderly patients that you see? (rank from 4 = most to 1 = least)**

| ❑ | Knowledge of medication frequency, dosage, and indication |
| --- | --- |
| ❑ | Skills to open bottles, read labels, and distinguish colors |
| ❑ | Difficulty with swallowing pills or capsules |
| ❑ | Polypharmacy |

1. **Which of the following interventions do you find with your patients is most effective in improving medication adherence in patients with memory loss or forgetfulness? (rank from 4 = most to 1 = least)**

| ❑ | Decreasing the total number of medications |
| --- | --- |
| ❑ | Simplifying dosing with combination therapy |
| ❑ | Using memory cues, pill boxes, electronic reminders |
| ❑ | Simplifying dosing with once-daily dosing |

1. **Which of the following factors is the strongest predictor of poor medication adherence? (select only one)**

| ❑ | Low reading ability |
| --- | --- |
| ❑ | Low annual income |
| ❑ | Severity of disease |
| ❑ | Subcutaneous rather than oral medication dosing |

**Case #3:** A 50 yr old asymptomatic white male came presented to the executive physical program for risk assessment of coronary disease. He denied history of hypertension, diabetes mellitus and smoking. Lipid profile was not known. His both parents died of “heart attack” in their sixties. He was taking vitamin E and folic acid. His diet and exercise were erratic due to a busy travel schedule. Physical examination: Height 5 foot 8 inches, weight 172 lbs, BP 170/94 mmHg, normal carotid and cardiac exam. As part of executive physical he had an exercise stress test which was normal. Lbs were: total cholesterol 210 mg/dl, LDL 130mg/dl, HDL 36mg/dl, Triglycerides 256mg/dl, fasting blood sugar 140mg, ALT 34(5-45) and urinalysis was normal.

1. **What is his LDL goal? (select only one)**

| ❑ | LDL<130mg/dl |
| --- | --- |
| ❑ | LDL < 100mg/dl |
| ❑ | LDL< 70mg/dl |

1. **Which of the following is incorrect: (select only one)**

| ❑ | Lifestyle change only (Diet, exercise and weight loss) |
| --- | --- |
| ❑ | Lifestyle change, statin and aspirin |
| ❑ | Lifestyle change, statin, niacin and aspirin |
| ❑ | Lifestyle change, statin, niacin, aspirin, continue folic acid and vitamin E |

# Case #4: A 78 year old woman presents to the office for establishment of care. She has recently moved in with her daughter but has not seen a physician in almost 2 years. She states she was told she had some elevated blood sugars, mild hypertension and was told to eat fewer sweets, and less red meat and eggs. She is a lifelong non-smoker. She enjoys bacon and eggs every morning with her new bridge partners, but passes on the pastries as she is concerned about diabetes. She provides a history of being able to walk to the grocery store a few years ago, but has not been doing this over the last few years. Vital signs include a blood pressure of 159/78, heart rate of 78 bpm, and a BMI of 29 kg/m^2^. The labs you ordered for this visit include an LDL of 199 mg/dL, TG of 479 mg/dL and a HbA1c of 6.0 %.

1. **What would be your initial approach for this patient? (select only one)**

| ❑ | Lifestyle and dietary modification. |
| --- | --- |
| ❑ | Lifestyle and dietary modification and treatment with a thiazide diuretic. |
| ❑ | Lifestyle modification and treatment with a statin. |
| ❑ | Lifestyle and dietary modification and treatment with both a thiazide diuretic and a statin. |

1. **Is this woman at risk for a cardiovascular event? (select only one)**

| ❑ | Yes |
| --- | --- |
| ❑ | No |

1. **When would you order a stress test on this woman? (select only one)**

| ❑ | I would order one as a follow up to today’s visit. |
| --- | --- |
| ❑ | I would order one today and yearly thereafter. |
| ❑ | I would order a test if she develops symptoms of chest pain, shortness of breath or atypical angina. |
| ❑ | I would not order a stress test on this elderly woman. |

1. **Please rate each of the following barriers to the optimal management of cardiovascular risk in your patients:**

**Not a barrier**  **Significant barrier**

**Adverse effects of drugs** 1 2 3 4 5

**Patient adherence** 1 2 3 4 5

**Presence of co-morbid conditions** 1 2 3 4 5

**Cost of medications** 1 2 3 4 5

**Number of drugs needed for** 1 2 3 4 5

**adequate blood pressure control**

**Patient understanding of treatment goals** 1 2 3 4 5

1. **Please rate your level of agreement with each of the potential practice barriers for you and your staff in the optimal management of cardiovascular risk in your patients.**

**Disagree**  **Agree**

**My practice has:**

**Adequate time to address lifestyle issues with** 1 2 3 4 5

**my patients**

**Adequate patient-education tools regarding** 1 2 3 4 5

**lifestyle issues**

**Knowledge and skills to provide** 1 2 3 4 5

**dietary recommendations**

**Knowledge and skills** 1 2 3 4 5

**to facilitate patient adherence**

# How confident are you in your ability to prevent and manage patients with cardiovascular disease? (circle only one)

**1               2 3 4   5        6              7     8 9 10**

not confident at all                    somewhat confident             very confident

**16. Overall, how useful do you find CME programs in keeping up-to-date in your practice?**

1 2 3 4 5 6 7 8 9 10

**Not at all useful Extremely useful**

**17. Please rank the following in terms of their importance in helping you provide optimal care to your patients.** (1 = most important, 4 = least important)

| ___ | Journal articles with new trial data |
| --- | --- |
| ___ | Opinions of experts and colleagues |
| ___ | Clinical practice guidelines |
| ___ | CME activities |

# 18. Approximately how many patients do you see each week? __________________/per week

# 19. Approximately what percentage of your patients has dyslipidemia? _________________%

# 20. Approximately what percentage of your patients has hypertension? _________________%

**21. How many years have you been in practice? ____________**

**22. How many physicians are in your practice (including yourself)?________________**

| **23. Physician Specialty** | **24. Practice Type** | **25. Practice Location** |
| --- | --- | --- |
| ❑ Family Practice | ❑ Private | ❑ Urban |
| ❑ Internal Medicine | ❑ Hospital | ❑ Suburban |
| ❑ General Practice | ❑ Academic | ❑ Rural |
| ❑ Other_______________________ | ❑ Non-practicing |  |
|  | ❑ Other____________________ |  |
